# Dietary modulation for the hypertension risk group in Koreans: a cross-sectional study

**DOI:** 10.1186/s12986-025-00921-4

**Published:** 2025-04-10

**Authors:** Youngmin Han, Ryun Huh, Keum Ji Jung, Heejin Kimm, Sun Ha Jee

**Affiliations:** 1 Institute for Health Promotion, Graduate School of Public Health, Yonsei University, Seoul, Republic of Korea; 2 Department of Epidemiology and Health Promotion, Graduate School of Public Health, Yonsei University, Seoul, Republic of Korea

**Keywords:** Hypertension, Hypertension risk factor, Principal component analysis, Dietary patterns, Nutritional moderation, Moderation effect analysis

## Abstract

**Background:**

Hypertension (HTN) is a critical global health issue, contributing to high morbidity and mortality rates. Representative risk factors for HTN include aging, genetics, obesity, alcohol drinking, smoking, and diet. Dietary interventions like the Dietary Approaches to Stop HTN (DASH) diet plan effectively prevent and manage HTN. We intend to evaluate the influence of eating patterns on HTN, applying multiple risk factors.

**Methods:**

For cross-sectional design, study subjects were grouped into four groups: optimal (*n* = 7,712), normal (*n* = 1,220), high normal (*n* = 3,655), and HTN (*n* = 4,355) according to the 2022 HTN treatment guidelines of Korea. Factor analysis was performed to identify major dietary patterns based on nutritional data obtained from a brief dietary questionnaire, including 17 food items. Finally, we conducted a moderation analysis to evaluate the impact of dietary patterns on the HTN risk score, which is determined by genetic variables, body mass index, alcohol consumption, and smoking status.

**Results:**

We identified three principal dietary patterns (Korean, Western, and New diet) in the study population. Adherence to the New diet was linked to lower HTN risk in all models (*p* < 0.001), while the Western and Korean diets were associated with a higher risk of HTN in some models. In high HTN-risk individuals, adherence to the Western diet increased the HTN risk trend (*p* < 0.001), whereas the New diet showed a potential protective trend (*p* = 0.059).

**Conclusions:**

The nutritional moderation effect was evident in the HTN high-risk group, where the Western diet increased risk, while the New diet showed a borderline protective effect. If the findings are validated by longitudinal investigation, our findings could serve as the basis for developing dietary guidelines for HTN.

**Supplementary Information:**

The online version contains supplementary material available at 10.1186/s12986-025-00921-4.

## Introduction

Hypertension (HTN), commonly known as high blood pressure, is a major global public health issue. The World Health Organization Global Report 2023 emphasizes the demand for improved HTN awareness and management, reporting a thirty-year rise in adults with HTN in Europe/Americas and South-East Asia/Western Pacific [1]. It is a leading risk factor for cardiovascular disorders (CVD), including heart attacks and strokes, which account for a sizable fraction of global deaths [1, 2]. Recent statistics indicate that approximately 30% of Korean adults suffer from HTN, with a higher prevalence among men than women [3].

There are non-modifiable and modifiable risk factors for HTN. Non-modifiable risk factors include age, as blood pressure tends to rise with aging, and genetic predisposition, where individuals with a family history of HTN are more at risk [4, 5]. In addition, males are more susceptible to HTN in middle age, but postmenopausal women are at a higher risk later in life. Modifiable factors, on the other hand, include unhealthy dietary habits, particularly high sodium intake, which is a leading cause of elevated blood pressure [6]. Excessive alcohol consumption and smoking constrict blood vessels, contributing to HTN [7]. A recent multivariable Mendelian randomization study observed that genetically predicted smoking and alcohol consumption behaviors interact synergistically, affecting HTN and other CVD, implying that those who both smoke and drink alcohol have a higher cumulative risk of disease [8]. Further, obesity and a sedentary lifestyle increase the workload on the heart, raising blood pressure [9, 10].

Dietary habits are critical in developing and managing HTN [11, 12]. In particular, numerous studies have shown a clear association between high sodium consumption and increased risk of HTN [13]. In contrast, diets rich in fruits, vegetables, whole grains, and low-fat dairy products, such as the Dietary Approaches to Stop HTN (DASH) diet, effectively lower blood pressure [14, 15]. In South Korea, traditional diets, which include a high amount of salt from meals such as Kimchi and fermented sauces, are thought to raise the risk of HTN [16]. However, studies examining the association with high blood pressure using dietary factors other than salt in the traditional Korean diet are still limited [17]. Furthermore, Korean eating patterns have changed throughout time [18, 19]. Consequently, the effect of changing eating habits on high blood pressure should be investigated, and nutritional strategies for HTN in Koreans should be recommended.

In the current cross-sectional research, we analyzed a large sample of Koreans from the Korean Cancer Prevention Study (KCPS)-II, demonstrating the effect of major dietary patterns on HTN risk groups characterized by genetic predisposition, obesity, alcohol consumption, and smoking status.

## Materials and methods

### Study population

Study subjects were selected from the KCPS-II cohort. The KCPS-II participants were registered from April 2004 through 18 health promotion centers distributed around South Korea. All participants gave written informed consent to participate in the cohort and use their data in secondary research. The Institutional Review Board at the Yonsei University Health System approved the study protocol under the Helsinki Declaration (IRB number: 4-2011-0277).

Among them, individuals aged 20–85 years who responded to a dietary questionnaire were selected for the present research. HTN was defined according to the 2022 HTN treatment guidelines of Korea [20]. Subjects with systolic blood pressure (SBP) < 120 mmHg and diastolic blood pressure (DBP) < 80 mmHg were categorized into the optimal group.

The normal group had SBP 120–129 mmHg and DBP < 80 mmHg, whereas those with SBP 130–139 mmHg and DBP 80–89 mmHg were classified as the high normal group. The HTN group included subjects with SBP ≥ 140 mmHg, DBP ≥ 90 mmHg, or taking antihypertensive medications. Participants reporting implausible energy intake of more than 5000 kcal/d or less than 500 kcal/d on dietary assessment were excluded. At last, the subjects were grouped into four categories: optimal (*n* = 7,712), normal (*n* = 1,220), high normal (*n* = 3,655), and HTN (*n* = 4,355) (Figure **S1**).

### Data collection

Each participant answered a self-administered questionnaire regarding sociodemographic characteristics and health habits, such as their alcohol consumption (never-drinker, ex-drinker, or current drinker) and smoking habits (never-smoker, ex-smoker, or current smoker). Clinical variables were obtained by anthropometric measurements and human specimens analysis in the hospital laboratory using a COBAS INTEGRA 800 and a 7600 Analyzer (Hitachi, Tokyo, Japan). Details on the examination methods are previously described [21, 22].

### Dietary assessment

In this study, the brief dietary questionnaire included 17 food items from 7 food groups: (1) fish, meat, eggs, and soybean products; (2) milk and dairy products; (3) vegetables; (4) fruits; (5) grains and potatoes; (6) sugars and candies; and (7) fats and oil [21]. Subjects were interviewed by a trained dietitian, who used food models and measuring instruments to estimate portion sizes according to the list of food exchanges for Koreans (3rd edition developed by the Korean Diabetes Association, the Korean Nutrition Society, the Korean Society of Community Nutrition, the Korean Dietetic Association, and the Korean Association of Diabetes Dietetic) [23]. Participants were instructed to indicate the frequency of their current consumption for each food item using four predefined categories: 0 (never), 0.5 (often), 1.0 (regular), and 1.5 (always sufficient). The intake amount of each food group was estimated based on the reported frequency of consumption and portion sizes.

The validity of this brief dietary assessment was evaluated in the previous study by comparing them with those measured by the 3-day diet records [21]. In that study, the correlation of energy, carbohydrate, and protein, was in reasonable validity (*r* > 0.4; *p* < 0.05), whereas those of fat and most minerals and vitamins were relatively low. In the current study, rather than focusing on the value of each nutrient, collected food items data was used to investigate dietary patterns associated with HTN.

Dietary patterns were identified using a principal component factor analysis based on food items, which were reclassified into nine food groups: vegetables, Kimchi, grains (rice, bread, and noodles), eggs, oil, fruit, dairy products (milk and yogurt), sugar (sugar, honey, and jam) and mixed protein sources (fish, meat, and bean products). The primary limitation of the dietary questionnaire is that fish, meat, and legumes were all covered in one question. Previous research excluded this group from the analysis due to interpretability issues [21], but we included it. Also, given that the objective of this study was HTN, it was unfortunate that the amount of sodium intake could not be determined.

### Genome-wide association study (GWAS) and genetic risk score (GRS)

DNA was genotyped using the KORV 1.0–96 Array (Affymetrix, Santa Clara, CA, USA) provided by the K-CHIP consortium and Affymetrix Genomewide Human single nucleotide polymorphism (SNP) Array 5.0 (Afymetrix Inc.).

For quality control, we excluded markers with a high missing rate (> 5%), individuals with a high missing rate (> 5%), and SNPs with minor allele frequency < 0.05 or a substantial divergence from Hardy-Weinberg equilibrium (*p* < 1.0 × 10^− 6^). The GWAS on HTN was carried out with PLINK 2.0. Logistic regression was performed to assess the impact magnitude of each SNP on HTN, adjusting for age and gender. The dependent variable was HTN status, coded as 1 for the HTN group and 0 for non-HTN (including individuals in the optimal, normal, and high normal groups).

After clumping (kb, 1000; p1, 5 × 10^− 7^; p2, 1.0 × 10^− 5^; r^2^, 0.3), 7 SNPs with *p* < 5.0 × 10^− 8^ through Bonferroni correction (Table **S1**) were used for calculating GRS in combination with the regression coefficient as a weight. Figure **S2** depicts the Manhattan plot and distribution of GRS.

### HTN risk score

The HTN risk score was calculated only in subjects with all values of body mass index (BMI), smoking status, alcohol consumption, and genetic risk scores [total (*n* = 16,748); optimal (*n* = 7,621), normal (*n* = 1,204), high normal (*n* = 3,630), and HTN (*n* = 4,293)] (Figure **S1**).

**Fig. 1:**
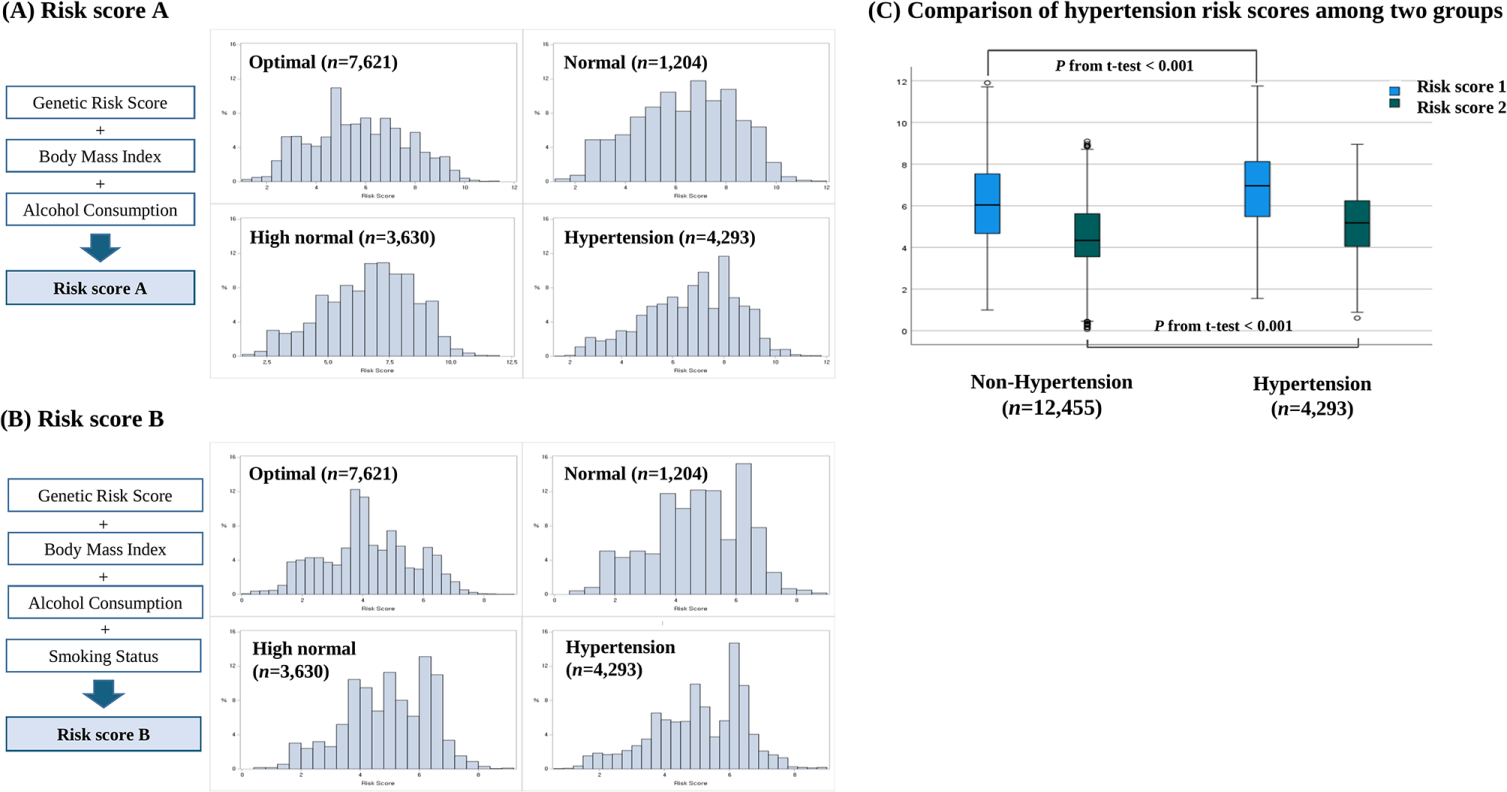
Calculation and distribution of hypertension risk score. (**A**, **B**) The components of hypertension risk score A and B are described. The histogram represents the distribution of risk scores for each group. (**C**) The comparison of risk scores between the HTN and non-HTN (optimal, normal, and high normal) groups was conducted, with the *p*-value calculated from t-test

At first, BMI, alcohol consumption, and smoking habits were converted to each score by multiplying the weights of the logistic regression model after the age and gender adjustment. The dependent variable was HTN status (non-HTN for those in the optimal, normal, and high normal groups vs. HTN). Two HTN risk scores were calculated. Contrary to common understanding, the beta value for the smoking variable in our research was 0.934, resulting in a risk score excluding smoking status (HTN risk score A). The scores of genetic risk, BMI, alcohol consumption, and smoking status were then summed together to calculate HTN risk score B (Fig. 1).

### Statistical analysis

All statistical analyses were performed using SAS 9.4 (SAS Institute, NC, USA) and SPSS 28 (IBM Corp, Armonk, NY, USA). T-test, ANOVA, and the Bonferroni correction among groups analyzed continuous variables. A Chi-squared test tested nominal variables. The data are expressed as the mean ± standard deviation, and two-tailed *p* values less than 0.05 were considered to indicate significance.

Principal component analysis was used to identify the primary dietary patterns based on nine food groups. We evaluated the adequacy and suitability of our sampling of the factor analysis using Kaiser–Meyer–Olkin (KMO) and Bartlett’s sphericity test. Applying varimax rotation, we derived specific dietary patterns closely connected to each food category [23]. Each dietary pattern has been designated a Korean, Western, or New diet based on the primary food categories following the previous study [21].

Logistic regression analyses were performed to assess the impact of dietary patterns and the magnitude of each SNP on categorical HTN status (HTN presence vs. non-HTN). The moderation effect analysis, adjusted for age, assessed how diet influences HTN risk. In this analysis, the independent variable (X) was the HTN risk score (continuous variable), and the moderator (M) was the diet pattern score (continuous variable). The dependent variable (Y) was the categorical HTN status (HTN presence vs. non-HTN), with an interaction term (X*M) included to examine moderation effects.

## Results

### Characteristics of the study population

Of 16,942 participants, 25.7% (*n* = 4,355) had HTN; additionally, 21.6% (*n* = 3,655) were in a high normal state (Table 1). The mean value of age was the highest in the HTN group (optimal, 44.8 ± 9.9; normal, 48.7 ± 11.7; high normal, 47.4 ± 10.4; HTN, 53.2 ± 10.1). Except for the optimal group, the other three groups had a greater proportion of males (optimal, 45.1%; normal, 60.7%; high normal, 72.1%; HTN, 71.1%). Regarding modifiable factors, 22.8% of the HTN group were current smokers, and 70.3% were current alcohol drinkers. In contrast, 22.7% of the HTN group were current smokers, and 64.2% were current alcohol drinkers.

**Table 1:** Baseline clinical and biochemical characteristics of subjects

	Optimal (*n* = 7,712)			Normal (*n* = 1,220)			High Normal (*n* = 3,655)			Hypertension (*n* = 4,355)			*P* value
Age (year)	44.8		± 9.9 ^a^	48.7		± 11.7 ^b^	47.4		± 10.4 ^c^	53.2		± 10.1 ^d^	**< 0.001**
Male *n*, (%)	3,481		(45.1)	740		(60.7)	2,634		(72.1)	3,096		(71.1)	**< 0.001**
Smoking status *n*, (%)													
Non-smoker	4,458	(57.8)		608	(49.8)		1,525	(41.7)		1,844	(42.3)		**< 0.001**
Ex-smoker	1,504	(19.5)		306	(25.1)		1,121	(30.7)		1,519	(34.9)		
Current smoker	1,750	(22.7)		306	(25.1)		1,009	(27.6)		992	(22.8)		
Alcohol consumption *n*, (%)													
Non-drinker	2,370	(31.0)		364	(30.2)		817	(22.5)		1,151	(26.8)		**< 0.001**
Ex-drinker	363	(4.8)		54	(4.5)		109	(3.0)		126	(2.9)		
Current drinker	4,900	(64.2)		787	(65.3)		2,708	(74.5)		3,022	(70.3)		
Body mass index (kg/m^2^)	22.9		± 2.8 ^a^	24.4		± 2.9 ^b^	24.5		± 3.0 ^b^	25.2		± 2.9 ^c^	**< 0.001**
Waist circumference (cm)	77.8		± 8.7 ^a^	82.3		± 8.5 ^b^	83.8		± 8.7 ^c^	86.1		± 8.4 ^d^	**< 0.001**
Systolic blood pressure (mmHg)	106.7		± 7.8 ^a^	123.4		± 2.7 ^b^	123.0		± 7.9 ^b^	134.0		± 14.7 ^c^	**< 0.001**
Diastolic blood pressure (mmHg)	68.5		± 7.0 ^a^	73.2		± 5.4 ^b^	82.8		± 4.2 ^c^	87.7		± 11.2 ^d^	**< 0.001**
Glucose (mg/dL)	88.0		± 15.6 ^a^	92.0		± 17.1 ^b^	93.9		± 21.9 ^c^	97.8		± 23.5 ^d^	**< 0.001**
Total cholesterol (mg/dL)	184.9		± 32.2 ^a^	191.6		± 35.1 ^b^	194.3		± 34.0 ^b^	194.2		± 34.4 ^b^	**< 0.001**
Triglyceride (mg/dL)	116.2		± 72.9 ^a^	142.0		± 103.9 ^b^	153.0		± 99.4 ^c^	165.2		± 106.9 ^d^	**< 0.001**
HDL-cholesterol (mg/dL)	54.8		± 13.1 ^a^	51.6		± 12.0 ^b^	51.3		± 12.1 ^b^	50.2		± 11.9 ^c^	**< 0.001**
LDL-cholesterol (mg/dL)	112.0		± 28.8 ^a^	118.2		± 31.4 ^b^	120.5		± 30.2 ^b^	119.1		± 30.8 ^b^	**< 0.001**
AST (IU/L)	21.4		± 13.1 ^a^	23.2		± 9.9 ^b^	24.1		± 25.4 ^b^	25.2		± 16.1 ^c^	**< 0.001**
ALT (IU/L)	21.4		± 20.3 ^a^	25.7		± 19.1 ^b^	28.2		± 49.1 ^b^	28.9		± 31.1 ^b^	**< 0.001**
GGT (IU/L)	26.7		± 28.4 ^a^	36.1		± 43.2 ^b^	41.9		± 63.8 ^c^	47.6		± 69.2 ^d^	**< 0.001**

Mean ± standard deviation (SD). Continuous variables were analyzed by ANOVA among groups. The same letters indicate non-significant differences between groups based on the Bonferroni correction

Nominal variables were tested by a Chi-squared test. Values in bold are significant at *p* < 0.05. HDL: high-density lipoprotein. LDL: low-density lipoprotein. AST: aspartate aminotransferase. ALT: alanine aminotransferase. GGT: γ-glutamyltransferase

The ANOVA on four groups (optimal vs. normal vs. high normal vs. HTN) indicates the statistical differences of all observed clinical variables (*p* < 0.001). Among them, BMI, waist circumference, SBP, DBP, glucose, total cholesterol, triglyceride, low-density lipoprotein-cholesterol, aspartate aminotransferase, alanine aminotransferase, and γ-glutamyltransferase tend to increase toward HTN (lowest in the optimal group and highest in the HTN group). However, the level of lower high-density lipoprotein-cholesterol was lower in the HTN group than in other groups.

### Nutritional data and major dietary patterns of the study population

Table 2 summarizes the nutritional data obtained from a brief dietary assessment. Energy intake (kcal) was significantly higher in HTN compared to other groups (*p* < 0.001). The ANOVA, which compared the daily intakes of 9 food groups (g/d) in four groups (optimal vs. normal vs. high normal vs. HTN), also showed statistical significance. Among them, grain and mixed protein sources tend to increase toward HTN (highest in the HTN group and lowest in the optimal group). On the other hand, milk exhibited the opposite trend.

**Table 2:** Distribution of nutritional data collected from a brief dietary assessment

	Optimal (*n* = 7,712)		Normal (*n* = 1,220)		High Normal (*n* = 3,655)		Hypertension (*n* = 4,355)		*P* value
**Energy (kcal)**	1760.7	± 327.2 ^a^	1847.6	± 346.5 ^b^	1849.0	± 331.0 ^b^	1865.0	± 334.4 ^b^	**< 0.001**
**Food groups (g/day)**									
Grain	613.8	± 155.1 ^a^	652.7	± 154.0 ^b^	644.9	± 156.2 ^b^	662.6	± 160.3 ^c^	**< 0.001**
Mixed protein sources	177.9	± 64.2 ^a^	190.1	± 74.9 ^b^	197.9	± 77.3 ^b^	198.6	± 76.9 ^c^	**< 0.001**
Egg	23.2	± 20.1 ^a^	22.3	± 21.0 ^a^	23.2	± 20.9 ^a^	21.4	± 21.7 ^b^	**< 0.001**
Vegetable	302.4	± 120.1 ^a^	315.8	± 123.2 ^a^	301.6	± 125.5 ^a^	315.9	± 122.6 ^b^	**< 0.001**
Kimchi	108.5	± 54.0 ^a^	114.7	± 55.5 ^b^	107.0	± 54.3 ^c^	113.2	± 55.6 ^b^	**< 0.001**
Fruit	241.7	± 199.0 ^a^	244.8	± 217.6 ^b^	231.5	± 187.0 ^b^	229.4	± 184.6 ^a^	**0.001**
Milk	128.7	± 129.0 ^a^	125.4	± 135.7 ^b^	118.5	± 121.3 ^c^	111.7	± 121.0 ^c^	**< 0.001**
Sugar	53.1	± 76.2 ^a^	56.6	± 85.3 ^b^	59.6	± 80.6 ^c^	50.0	± 70.8 ^c^	**< 0.001**
Oil	13.8	± 4.5 ^a^	13.6	± 4.2 ^b^	14.3	± 5.0 ^c^	13.8	± 5.9 ^c^	**< 0.001**

Mean ± standard deviation (SD). *P*-values were derived from ANOVA among groups. The same letters indicate non-significant differences between groups based on the Bonferroni correction test. Values in bold are significant at *p* < 0.05. The mixed protein sources group included meat, fish, and tofu

In a principal component analysis for identifying dietary patterns, the KMO index was analyzed as 0.595 with Bartlett’s test of *p* < 0.001, and three patterns (Korean diet, Western diet, and New diet) were derived. Those foods placed high on the factor loading on the Korean diet pattern include grain, mixed protein sources, vegetables, and Kimchi. The Western diet pattern is centered on sugar, eggs, and oil, whereas the New diet pattern is defined by a high consumption of fruits and dairy products and a low sugar intake (Table 3). Using the orthogonal rotation (varimax) factors, we calculated a score expressing adherence to each of the three dietary patterns.

**Table 3:** Rotated factor loadings for the food groups across the three dietary patterns extracted through principal component analysis

	Factor 1 Korean diet pattern	Factor 2 Western diet pattern	Factor 3 New diet pattern
Grain	0.682		
Mixed protein sources	0.526	0.409	
Egg		0.671	
Fruit			0.748
Vegetable	0.583		0.488
Kimchi	0.741		
Sugar		0.434	-0.374
Oil		0.700	
Milk			0.447

Values less than 0.300 were excluded for simplicity. The mixed protein sources group included meat, fish, and tofu

### Association between three major dietary patterns and HTN

A binary logistic regression was conducted to assess the effect of each dietary pattern on blood pressure outcomes categorized as HTN and non-HTN groups (Table **S2**).

In the crude model (model 1), the odds of HTN increased by 1.269 (95% CI [1.226 to 1.314], *p* < 0.001) for a one-unit increase in the Korean diet score. Age-, and gender-adjusted model (model 2) showed similar results; the odds of HTN increased by 1.105, 95% CI [1.064 to 1.148], *p* < 0.001). However, when additionally adjusting for BMI, alcohol consumption, and smoking status (model 3–5), the odds of HTN did not significantly increase for every unit increase in the Korean diet score.

Regarding the Western diet, the odds of HTN decreased by 0.967 for a one-unit increase in diet score (95% CI [0.934–1.001], *p* = 0.060). After age and gender adjustment, the probabilities of the HTN increased by 1.088 times for every unit rise in Western diet score (95% CI [1.047 to 1.130], *p* < 0.001). The significance of being HTN increased for every unit increase in the Western diet score disappeared when adding an adjustment for BMI, alcohol consumption, and smoking status (model 3–5).

The risks of being HTN decreased by 0.911 (95% CI [0.879 to 0.944], *p* < 0.001) for every one-unit rise in the New diet in the crude model. Moreover, the significance remained in all adjusting models (all for *p* < 0.001). These results suggest a favorable association between the New diet adherence and blood pressure.

### Risk score calculation for identifying high-risk HTN group

For GRS, the mean value for the non-HTN group was 0.24 ± 0.36 (mean ± SD), whereas the HTN group exhibited a slightly higher mean of 0.31 ± 0.35. Regarding risk score A, the mean score for the non-HTN group was 6.05 ± 1.95, while the HTN group showed a higher mean of 6.75 ± 1.80. Similarly, for risk score B, similar results were shown (non-HTN group, 4.44 ± 1.53 vs. HTN group, 5.05 ± 1.43). Furthermore, the differences in GRS, risk score A, and risk score B between the two groups (non-HTN vs. HTN) were statistically significant (*p* < 0.001) (Fig. 1, Figure **S2**).

### Effects of major dietary patterns in the HTN high-risk groups

**Fig. 2:**
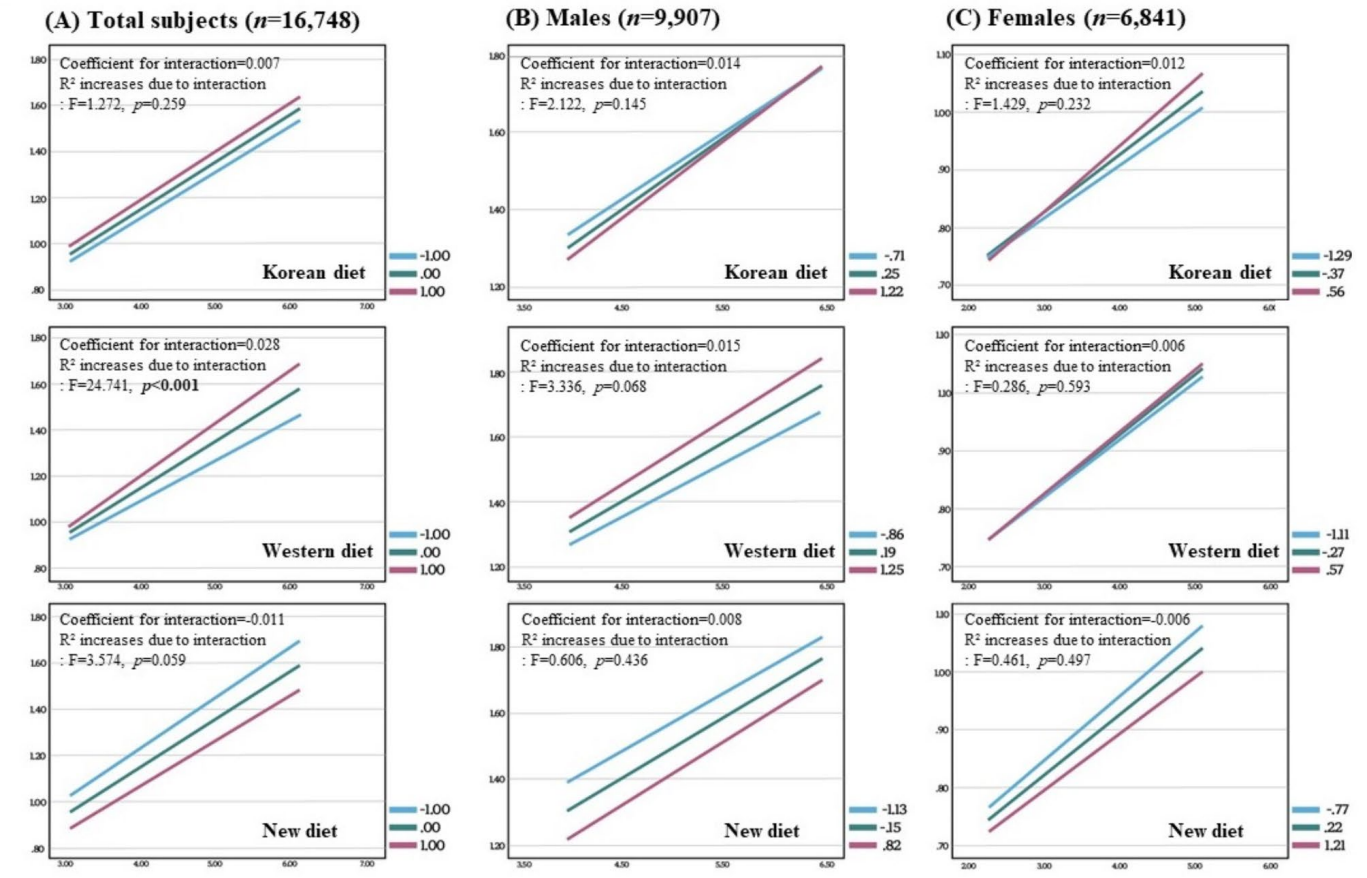
Moderation effect of dietary pattern on hypertension risk score **A**. Hypertension risk score A was calculated using genetic risk score, body mass index, and alcohol consumption. The moderation effect analysis was conducted with the hypertension risk score as an independent variable (X), each diet pattern score as moderator (M), whether hypertension or not as the dependent variable (Y), and X*M as the interaction term. A higher hypertension risk score means higher risk individuals for HTN. Red line: +standard deviation of M. Green line: mean. Blue line: -standard deviation of M. Coefficient for interaction: regression coefficient for the interaction effect between the X and M. R² increases due to interaction (F- and *p*-value): the metric indicating how much the explanatory power of the regression model improves when the interaction term is added to the model

Figure 2 indicates the results of the moderation effect analysis. It was confirmed that the R² was increased in the model, including the interaction term between the HTN risk score A and Western diet compared to the basic regression model in total subjects (coefficient for interaction = 0.028, F = 24.741, *p* < 0.001) and males (coefficient for interaction = 0.015, F = 3.336, *p* = 0.068). This means that individuals with a high HTN score may have a higher risk of developing HTN if their adherence to the Western diet is high. The effect was more significant in males than females.

Besides, in the regression model with the interaction term of HTN risk score A*New diet included, R² increased due to interaction (F = 3.574, *p* = 0.059). It suggests that the interaction between the New diet and HTN risk score A strongly predicts HTN compared to those without interaction. Because the interaction coefficient was − 0.011, those with a high HTN score could be at a decreased risk of HTN if they adhere to the New diet.

In the risk score model that includes smoking status (HTN risk score B), only the Western diet appears to significantly impact the development of HTN in high HTN risk groups (Figure **S3**).

## Discussion

This study concluded that the principal dietary patterns of Koreans (Korean, Western, and New diets) substantially influenced HTN prevalence. Based on the moderation effect analysis, we highlighted that the Korean diet had no discernible impact on the HTN risk in the HTN high-risk population. In contrast, the Western diet significantly increased the likelihood of developing HTN in high-risk populations, whereas the New diet reduced the risk (*p* = 0.059, with the statistical significance borderline). It is crucial as the score determining the HTN risk group considered a variety of risk factors for high blood pressure, including heredity, obesity, alcohol consumption, and smoking status. If the results are supported through longitudinal research, they can be the foundation for establishing dietary guidelines tailored to Koreans to prevent and manage HTN.

The calculated HTN risk score, composed of almost all known risk factors (genetic predisposition, BMI, alcohol consumption, and smoking status), reliably reflects HTN risk. Mapped genes with significant SNPs in the current study (WNT2B, DNAJC5B, ATXN2, ALDH2, and OAS1) are previously reported regarding association with HTN risk. WNT2B is critical in cardiovascular development and homeostasis involving the Wnt signaling pathway. Variants in this gene may influence blood pressure regulation by affecting vascular remodeling and endothelial function [24–26]. DNAJC5B is responsible for cellular stress responses and may contribute to HTN by regulating the survival and function of cardiac myocytes [27, 28]. ATXN2 has also been suggested to modulate HTN through multifaceted ways, with its involvement in inflammation, stress responses, and neuronal blood pressure regulation potentially affecting how signals relate to blood pressure regulation [29, 30]. ALDH2 is essential for aldehyde metabolism, and its variants are associated with alcohol-related HTN; Individuals with deficient ALDH2 activity may experience elevated blood pressure due to the accumulation of toxic aldehydes [31–33]. OAS1 is part of the immune response system and has been shown to influence inflammation, a known risk factor for HTN [34, 35]. Also, polymorphism of OAS1 was reported as a predisposing risk factor for Korean type 2 diabetes patients [36].

In general, individuals who smoke, consume alcohol, or are obese become more susceptible to HTN [7]. However, the current study found that smokers had a decreased risk of HTN than non-smokers. The explanation might be that smoking status data was gathered from self-reported data. Respondents may not properly evaluate their health state or habits, and there is a possibility of social desirability bias, which occurs when people underreport or overreport their behaviors, such as smoking status or alcohol consumption [37]. Another thing to consider is that this study is a cross-sectional design. Given that the data were collected at the same time, the participants may quit smoking after becoming aware of the onset of the disease. Therefore, in this study, two HTN risk scores, including and without smoking status, were calculated and used for the analysis. Further, longitudinal research is needed to consider the association between smoking and HTN accurately.

Results related to the Korean diet are interesting, as they provide different insights from previously known facts. A positive association between the Korean diet and HTN in the age- and gender-adjusted logistic model means that higher Korean diet pattern adherence is associated with higher HTN risk. However, the significance disappeared when other risk factors were added as an adjustment. Besides, the Korean diet did not significantly influence the HTN risk in the high HTN risk score group in the moderation effect analysis; the nonsignificant increase in R² due to the interaction between the HTN risk score and the Korean diet score indicated that the interaction effect did not meaningfully improve the explanatory power of the model for HTN (R² increases due to interaction of *p* > 0.05).

As the Korean diet represented by soup, Kimchi, and fermented food (ex., Gochujang) contains higher salt levels, it is considered a risk factor for HTN; our logistic model adjusting for age and gender also consistently supports this. Meanwhile, a recent animal study revealed that fermented food exerts antihypertensive effects, regardless of its high salt content, by regulating the renin-angiotensin-aldosterone system [38]. Beyond that, the Korean diet allows for consuming diverse food categories. Indeed, the Korean diet was classified with grain, mixed protein sources, vegetables, and Kimchi in the factor analysis of the present study. Pigmented and multigrain rice are rich in dietary fiber, magnesium, and antioxidants, which help decrease blood pressure [39]. Furthermore, whole grains have a more substantial favorable effect on blood pressure than refined grains [40]. This evidence suggests that multigrain rice, with higher nutrient density, can contribute to healthier blood pressure management, especially when consumed as part of a balanced diet. Also, since Kimchi, frequently consumed by Koreans, contains not only salt but also various vegetables as its main ingredient, we cannot state for certain that it has an unconditionally detrimental influence on HTN. From this perspective, further studies are needed to clarify the association between the Korean diet and HTN risk. Besides, in the principal component analysis, the rotated factor for mixed protein sources was highest in the Korean diet; the rotated factor for the Korean diet in our study was 0.526, and the Western diet was 0.409. This could reflect increased protein consumption among Koreans compared to the past [41]. However, it could also be a limitation arising from merging different protein sources into a single question, as the Western diet generally has higher animal protein intake [42]. Therefore, further research on the relationship between HTN and dietary patterns, including protein intake, is needed through more detailed data collection that specifies protein sources.

Western diet, including oil, sugar, and egg, appeared to worsen HTN risk. Especially in moderation effects analysis, R² increases due to the interaction between the Western diet and HTN risk score (F = 24.741, *p* < 0.001) in total subjects was significant. Previously reported results regarding the components of the Western diet are diverse. First, dietary oils have a complex relationship with blood pressure, presenting both risks and benefits. It was found that the degradation of frying oils can lead to increased HTN, inducing the harmful effects of oxidized fats on cardiovascular health [43]. Similarly, He et al. [44] employed latent class analysis to categorize participants based on their dietary intake patterns; they found that excessive oil and salt intake significantly contribute to high blood pressure, highlighting the need for moderation. In contrast, Massaro et al. [45] demonstrated that olive oil, particularly extra virgin, can lower blood pressure due to its polyphenol content. Interestingly, several studies contend that sugar is a more critical dietary factor than salt in contributing to HTN, as it can lead to insulin resistance and obesity [46, 47]. A clear link between sugar intake and increased risk of HTN based on a systematic review and dose-response meta-analysis supported this view [47]. The last one is an egg. Vu et al. [48]. analyzed nutritional data obtained through 24-hour dietary recalls and food frequency questionnaires in the U.S. population aged 40 to 59 years, suggesting that individual nutritional patterns and lifestyle factors may influence the effects of eggs, and Mesas et al. [49] indicated that the association varies based on BMI. However, a meta-analysis using mixed populations across various randomized control trials concluded that egg intake does not significantly affect blood pressure [50]. In conclusion, the negative effect of the Western diet on the risk of HTN, considering genetic traits, BMI, alcohol consumption, and smoking status shown in this study, could be valuable evidence.

The adherence to a New diet consisting of fruit, vegetables, milk, and low sugar was inversely associated with HTN risk in our study (*p* of all logistic models < 0.001). However, there was no significance in the moderation effect analysis (*p-*value with a statistical significance borderline). Recent studies underscore the link between fruit and vegetable consumption and reduced HTN risk. Kong et al. [51] reported that higher intakes of fruits, vegetables, and legumes are associated with reduced HTN risk among middle-aged and older Koreans. In that study [51], most subgroups exhibited inverse relationships, particularly among males with higher BMI. Various studies have reported consistent results [52, 53]. Notably, the New diet of our research seems to be similar to the DASH diet. The DASH eating plan recommends the consumption of fruits, vegetables, nuts/legumes/vegan protein, whole grains, low-fat dairy, and limiting the intake of sodium, red or processed meats, and sugar-sweetened drinks [15]. However, as protein sources are mixed in our dietary survey, it is difficult to estimate legumes, vegan protein intake, and the restriction of red or processed meats suggested by the DASH diet. Indeed, a rotated factor of mixed protein sources was 0.526 for the Korean diet, 0.409 for the Western diet, and < 0.300 for the New diet in our research, making it challenging to comprehend clearly. Hence, more detailed data collection is needed to establish dietary guidelines for preventing and managing HTN tailored to Koreans.

There are some limitations to the dietary questionnaire when applying it to HTN research. One major limitation is that three protein sources—meat, fish, and beans—were grouped in a single question, making it difficult to distinguish their individual effects on HTN. Animal and plant proteins have different effects on blood pressure; a diet high in animal and low in plant protein may raise blood pressure through increased BMI, whereas a plant-based diet is associated with lower blood pressure [54, 55]. A longitudinal study in China found that consuming less of both animal and plant protein, as well as total protein, was associated with a reduced risk of HTN [56]. Another important limitation is the absence of sodium intake data, which directly affects HTN risk. The study questionnaire did not allow for an accurate calculation of sodium intake, making it difficult to assess its contribution to HTN. The last one is that our brief questionnaire consisted of only 17 items. It could have lower reliability or accuracy than a more comprehensive FFQ or dietary record. However, this brief questionnaire was validated against 3-day food records in a prior study [21]. Furthermore, in the present study population, significant positive correlations were observed between total energy intake and both BMI (*r* = 0.259, *p* < 0.001) and waist circumference (*r* = 0.329, *p* < 0.001), suggesting that the dietary data retains reasonable reliability despite its limitations. Established validity from previous research and significant correlation with anthropometric measures (BMI and waist circumference) support its reliability, even though evaluating total caloric intake based only on the frequency and portion size of 17 items may overlook a significant portion of the diet and potentially compromise the accuracy of the dietary data. Although simplified, this questionnaire could efficiently capture key dietary information in large-scale population studies. To mitigate potential inaccuracies and provide a broader understanding of dietary influences on HTN, we focused on major dietary patterns rather than individual food items. This approach captures the interactions between food groups, offering a more comprehensive view of dietary habits and their impact on health [57].

Despite several limitations, it is noteworthy that we made a high-risk group in which risk variables were weighed in a large Korean population and evaluated the moderation impact of diet patterns on high-risk groups. Consequently, the nutritional moderation effect was evident in the HTN high-risk group, where the Western diet increased risk, while the New diet showed a borderline protective effect. To our knowledge, there are no comparable research designs on HTN among the Korean population. Collectively, this study pointed out that diet has a considerable impact on HTN risk. However, uncovering evidence by collecting dietary data through more detailed nutritional questionnaires on longitudinal study designs is necessary to develop evidence-based dietary guidelines such as the DASH eating plan.

## Electronic supplementary material

Below is the link to the electronic supplementary material.

Supplementary Material 1

Supplementary Material 2

Supplementary Material 3

Supplementary Material 4

Supplementary Material 5

## Data Availability

Some or all datasets generated and/or analyzed during the current study are not publicly available but can be made available by the corresponding author upon reasonable request.

## Abbreviations

BMI: Body mass index
CVD: Cardiovascular disorders
DASH: Dietary approaches to stop hypertension
DBP: Diastolic blood pressure
GRS: Genetic risk score
GWAS: Genome-wide association study
HTN: Hypertension
KCPS: Korean cancer prevention study
KMO: Kaiser–Meyer–Olkin
SBP: Systolic blood pressure
SNP: Single nucleotide polymorphism
