# Supplementary figures and images for: Dietary modulation for the hypertension risk group in Koreans: a cross-sectional study
Source: Nutr Metab (Lond). 2025 Apr 10;22:30. doi: 10.1186/s12986-025-00921-4 (PMC11987358; doi:10.1186/s12986-025-00921-4)

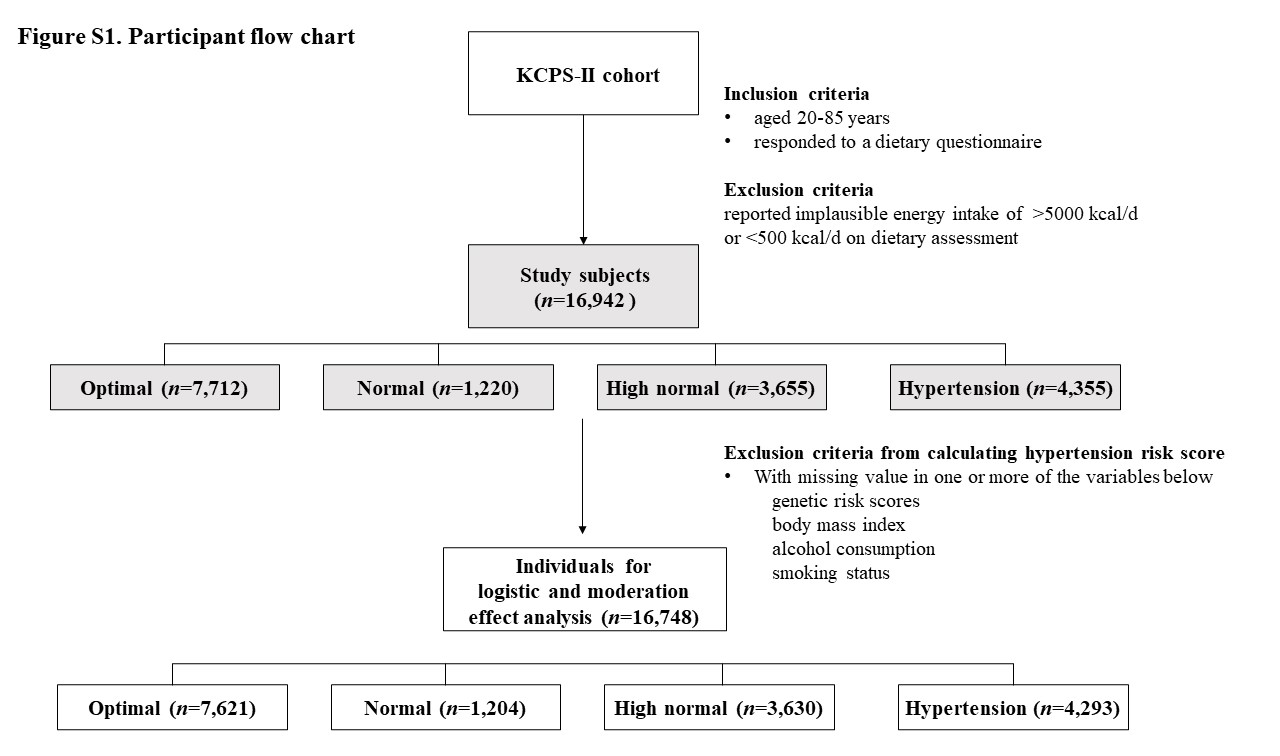

Supplement: Supplementary file 1 — Supplementary Material 1 [file 12986_2025_921_MOESM1_ESM.jpg]

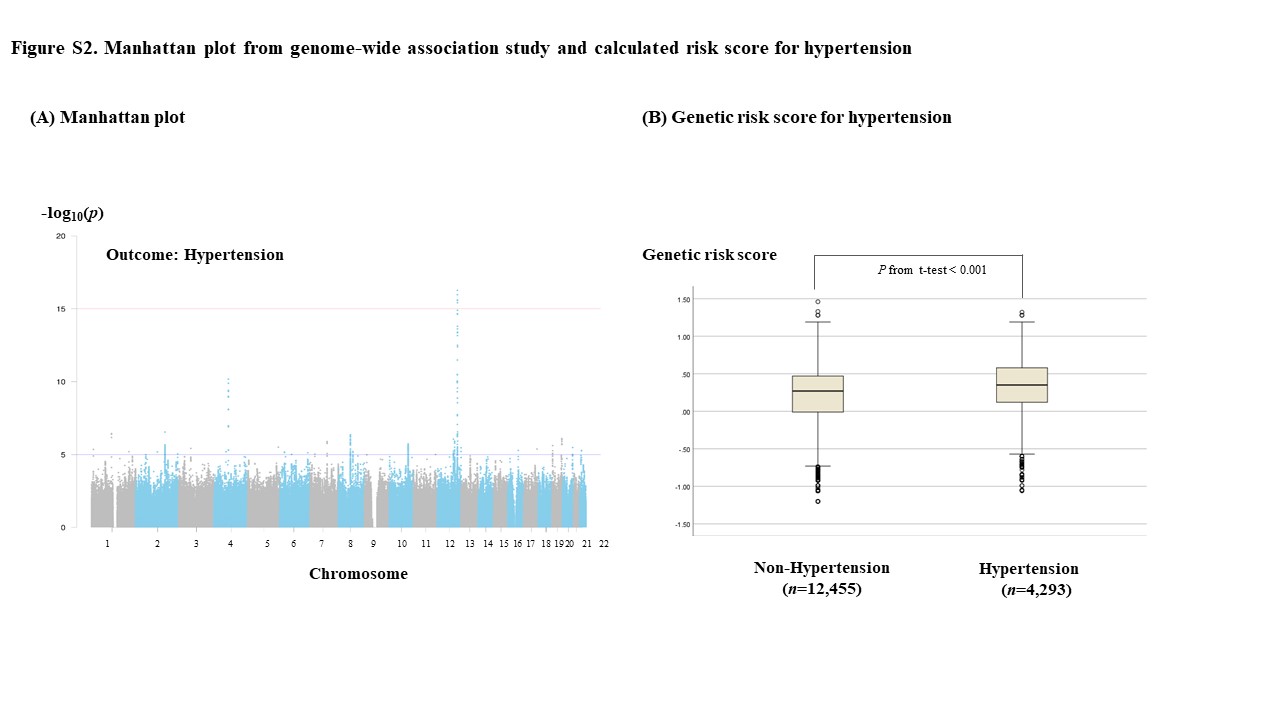

Supplement: Supplementary file 2 — Supplementary Material 2 [file 12986_2025_921_MOESM2_ESM.jpg]

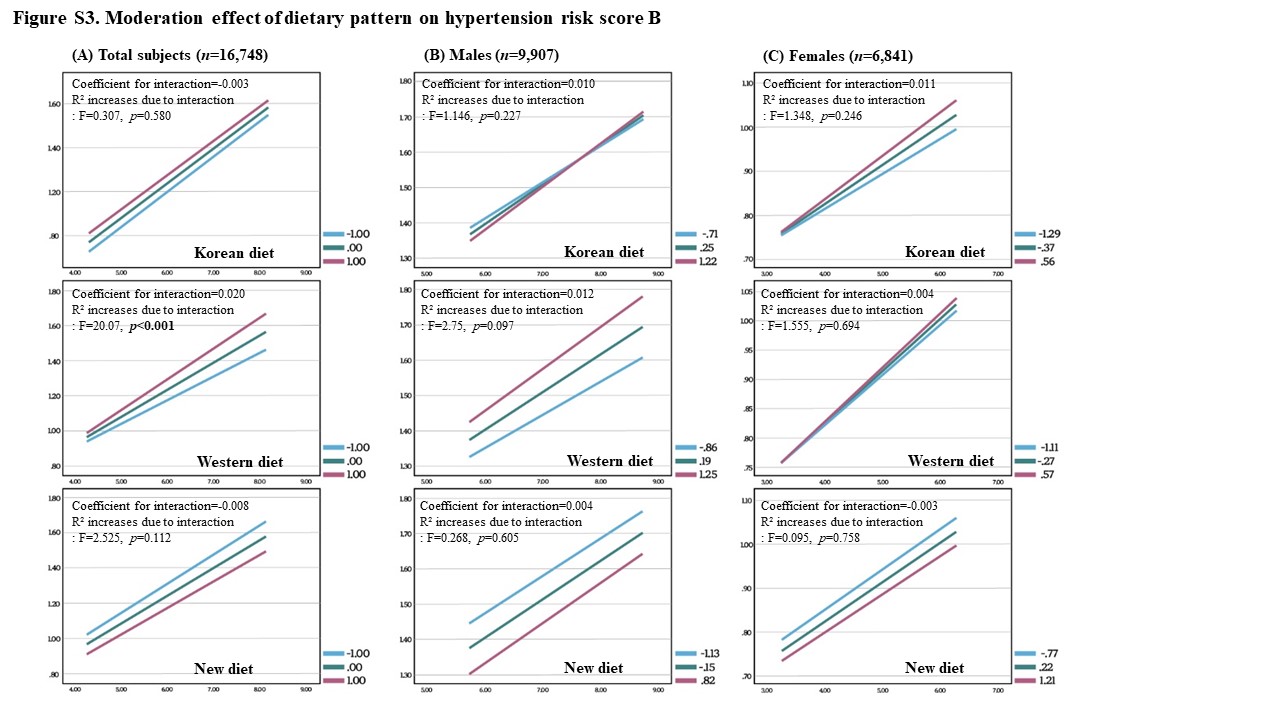

Supplement: Supplementary file 3 — Supplementary Material 3 [file 12986_2025_921_MOESM3_ESM.jpg]
