# Supplementary material for: Dietary modulation for the hypertension risk group in Koreans: a cross-sectional study
Source: Nutr Metab (Lond). 2025 Apr 10;22:30. doi: 10.1186/s12986-025-00921-4 (PMC11987358; doi:10.1186/s12986-025-00921-4)
Supplement: Supplementary file 4 — Supplementary Material 4 [file 12986_2025_921_MOESM4_ESM.docx]

**Supplementary Table 1. List of SNPs used in GRS calculation**

| **SNP** | **Position** | **Mapped Gene** | **Effect allele** | **OR** | **95 % CI** | ***P* from GWAS** |
| --- | --- | --- | --- | --- | --- | --- |
| rs10776752 | Chr1:112501706 | *WNT2B* | T | 1.156 | 1.093 - 1.223 | 3.66E-07 |
| rs268263 | Chr2:164097664 |  | T | 0.871 | 0.826 - 0.918 | 2.87E-07 |
| rs16998073 | Chr4:80263187 |  | T | 1.197 | 1.134 - 1.264 | 6.66E-11 |
| rs17398736 | Chr8:66036148 | *DNAJC5B* | G | 1.315 | 1.182 - 1.462 | 4.40E-07 |
| rs11065933 | Chr12:111504689 | *ATXN2* | C | 0.864 | 0.819 - 0.911 | 8.74E-08 |
| rs671 | Chr12:111803962 | *ALDH2* | A | 0.733 | 0.682 - 0.789 | 5.53E-17 |
| rs11066453 | Chr12:112927816 | *OAS1* | G | 0.813 | 0.751 - 0.880 | 2.76E-07 |

Significant associations from Genome-wide association analysis (*p* ≤ 5.0 x10^-8^) were presented. OR: Odds ratio. CI: confidence interval.
