# Supplementary material for: Dietary modulation for the hypertension risk group in Koreans: a cross-sectional study
Source: Nutr Metab (Lond). 2025 Apr 10;22:30. doi: 10.1186/s12986-025-00921-4 (PMC11987358; doi:10.1186/s12986-025-00921-4)
Supplement: Supplementary file 5 — Supplementary Material 5 [file 12986_2025_921_MOESM5_ESM.docx]

**Supplementary Table 2. Association between dietary patterns and hypertension**

|  | **Korean dietary pattern** | | | **Western dietary pattern** | | | **New dietary pattern** | | |
| --- | --- | --- | --- | --- | --- | --- | --- | --- | --- |
|  | **OR** | **95 % CI** | ***P* - value** | **OR** | **95 % CI** | ***P* - value** | **OR** | **95 % CI** | ***P* - value** |
| Model 1 | **1.269** | 1.226 - 1.314 | **<0.001** | 0.967 | 0.934 - 1.001 | 0.060 | **0.911** | 0.879 – 0.944 | **<0.001** |
| Model 2 | **1.105** | 1.064 - 1.148 | **<0.001** | **1.088** | 1.047 - 1.130 | **<0.001** | **0.894** | 0.860 – 0.929 | **<0.001** |
| Model 3 | 1.027 | 0.987 - 1.068 | 0.188 | 1.026 | 0.986 - 1.068 | 0.199 | **0.902** | 0.867 – 0.938 | **<0.001** |
| Model 4 | 1.030 | 0.990 - 1.071 | 0.148 | 1.028 | 0.988 - 1.070 | 0.168 | **0.902** | 0.867 – 0.938 | **<0.001** |
| Model 5 | 1.028 | 0.988 - 1.070 | 0.167 | 1.033 | 0.992 - 1.075 | 0.115 | **0.894** | 0.859 - 0.930 | **<0.001** |

This analysis was conducted on the 4,355 individuals diagnosed with hypertension and 12,587 individuals without hypertension. Model 1 was crude; Model 2 was adjusted for age and gender; Model 3 was adjusted for age, gender, and BMI; Model 4 was adjusted for age, gender, BMI and alcohol consumption; Model 5 was adjusted for age, gender, BMI, alcohol consumption, and smoking. *P* values were derived from logistic regression. Values in bold are significant at *p*<0.05. OR: odds ratio. CI : confidence interval.
